# Supplementary material for: Room-temperature photonic logical qubits via second-order nonlinearities
Source: Nat Commun. 2021 Jan 8;12:191. doi: 10.1038/s41467-020-20417-4 (PMC7794483; doi:10.1038/s41467-020-20417-4)
Supplement: Supplementary file 1 — Supplementary Information [file 41467_2020_20417_MOESM1_ESM.pdf]

Supplementary Information - Room-Temperature Photonic Logical Qubits via Second-Order Nonlinearities

Krastanov et al.

(Dated: November 1, 2020)

In these supplementary materials we give more details about the literature survey we have performed in order to estimate the feasibility of our design. We also elaborate on the performance of our numerical search techniques.

Supplementary Note 1 - Mapping between the modes of the processor and the modes of the error correcting code

In Supplementary Figure 1 we provide a more detailed rendition of how the two modes of the bosonic code we consider are passed through the nonlinear resonators. Notice that a resource of significant importance in this scheme is the “reshuffling” of modes. For instance, the second column of gates routes the code modes (in red and purple) to different spatial modes, depending on whether an ancillary photon was present.

Supplementary Note 2 - Additional examples of multiphoton operations

For working with single- and dual-rail encodings, we can use linear optics and the already described Toffoli gate. CPhase gates, being similar, but conceptually simpler than Toffoli gates, are also available. When considering higher photon states, our design can be used for additional operations in state preparation, control and measurement. Consider the following application for example:

Binary Decomposition Gate An important advantage of our design is the ease with which it can work on higher-photon-number states. This is crucial if we want to employ bosonic codes or perform number-resolving measurements. Here we demonstrate a gate that transforms a Fock state containing up to four photons of a single mode, into a multi-mode Fock state that contains a binary representation of the initial number of photons. This gate maps $|000\rangle$ and $|001\rangle$ into themselves, and transforms the remaining states as

$$|002\rangle \mapsto |011\rangle \tag{1}$$

$$|003\rangle \mapsto |101\rangle \tag{2}$$

$$|004\rangle \mapsto |111\rangle. \tag{3}$$

This gate enables a deterministic photon-number resolving measurement to be made by a set of photodetectors that are not themselves number resolving. This would require only a logarithmic number of detectors, instead of the exponentially large requirements of typical beam splitter trees [1, 2]. Another application is running this gate in reverse in order to prepare interesting higher-photon-number states, e.g., code words of bosonic codes. Lastly, this gate can be used for heralded generation of high-fidelity Fock states by inputting a weak coherent state and performing post-selection measurements on a subset of the modes.

Supplementary Note 3 - The mapping between the abstract control pulses and the corresponding

Let us further consider the controllable part of the Hamiltonian. In the main text we have treated $p(t)$ as a dimensionless function. We will now connect it to the quadratures $q(t)$ of the classical field $\mathbf{D}_p(\mathbf{r},t) = q(t)\mathbf{d}_p(\mathbf{r}) + \text{c.c.}$ Following the same process as above, we derive

$$\hat{H}_p(t) = -\frac{\chi^{(2)}\hbar\sqrt{\omega_b\omega_c}}{\sqrt{\varepsilon_0}n^3\sqrt{V_{\text{twm}}}}\, q(t)\hat{b}^\dagger\hat{c} + \text{H.c.} \tag{4}$$

$$\frac{1}{\sqrt{V_{\text{twm}}}} = \frac{\int_{NL} d_p^i d_b^{j*} d_c^k \mathrm{d}\mathbf{r}}{\sqrt{\int |\mathbf{d}_p|^2 \mathrm{d}\mathbf{r} \int |\mathbf{d}_b|^2 \mathrm{d}\mathbf{r} \int |\mathbf{d}_c|^2 \mathrm{d}\mathbf{r}}}, \tag{5}$$

where V_{twm} is the mode volume for three-wave mixing. Therefore, the unit of the ordinate in Fig. 2 is $u_p = \sqrt{\frac{1}{8}\frac{V_{\text{tmw}}}{V_{\text{shg}}}\frac{\hbar\omega_a\omega_b}{\omega_c}}$ and the time dependent control field is

$$\mathbf{D}(\mathbf{r}) = p(t)u_p\mathbf{d}_p(\mathbf{r}) + \text{c.c.} \tag{6}$$

$$\int \frac{|\mathbf{d}_p|^2}{\varepsilon_0 n^2}\mathrm{d}\mathbf{r} = 1, \tag{7}$$

where $\mathbf{d}_p$ is the normalized eigenmode of the cavity. In other words, the average number of ω_p photons in the control field needs to be just $\frac{1}{4}\frac{V_{\text{tmw}}}{V_{\text{shg}}}\frac{\omega_a\omega_b}{\omega_c\omega_p}|p(t)|^2$. Importantly for design considerations, while a very low V_{shg} (i.e., a high

overlap between modes $\hat{a}$ and $\hat{b}$) is an indisputable requirement, one can employ a high V_{twm} (i.e., low overlap between the control mode at ω_p and modes $\hat{b}$ and $\hat{c}$) as long as higher power in the control mode can be tolerated by the material.

Supplementary Note 4 - Literature Survey of Second-harmonic-generation Experiments

In Supplementary Figure 2 we see the progress in second-harmonic-generation (SHG) over the last decade, showing 10 orders of magnitude improvement in measures of efficiency related to our design [3–25]. We see the substantial progress in Q factors of photonic mircroresonators. Lithium niobate is the material of choice due to its high $\chi^{(2)}$. Photonic crystals are promising thanks to their extreme mode confinement, but still need improvements in Q factors. Microrings and whispering gallery resonators hold the majority of high-performance spots for now thanks to a good balance between mode confinement and Q factors. A typical figure of merit in SHG experiments is the efficiency [24] $\eta = \frac{P_{\text{out}}}{P_{\text{in}}^2} \propto \frac{Q^3(\chi^{(2)})^2}{V_{\text{shg}}}$, which is closely related to the number-of-useful-operations figure of merit we are using in the main text. While it is infeasible to directly plot our figure of merit for the results above (due to the vastly diverse hardware in which they were obtained), the exponential growth of the easier-to-measure efficiency bodes well for the future use of our protocol. Note that recently the leading experiments have switched from whispering gallery resonators (orange) to better confined microring resonators (blue), even though their Q factors are much lower, leaving very significant space for further improvements.

Highly nonlinear materials can significantly improve the performance of our protocol. The progress in their development is depicted in Supplementary Figure 3. Significant improvements have been achieved both by the discovery of new materials and by placing known materials under strain using novel fabrication techniques. The survey of Pockels-effect electro-optical modulator hardware [26–39] shown here reveals the progress in the size of effective electro-optical coefficients for on-chip nonlinear optics. For comparison, the values for these coefficients in bulk crystals are shown in dashed lines. While not all of these results carry over to the optical regime due to frequency dependence, our design can make use of the low-frequency regime if electrical pulses are used to implement the control function $p(t)$. It is particularly interesting that advanced fabrication techniques are capable of inducing record-high $\chi^{(2)}$ values even in materials that do not have second order nonlinearities in their bulk form (e.g., strained SiN in [29]).

Supplementary Note 5 - Optimal Control Pulses

The main text established the following parameterization for the control pulse used to construct a given unitary operation $\hat{U}$:

$$\hat{U}(\mathbf{v}) = \prod_{l=1}^{s} \exp\left\{-i\left[\mathrm{f}(X_i, P_i)\hat{b}^\dagger\hat{c} + \sigma(T_i)\hat{a}\hat{b}^{\dagger 2} + \text{H.c.}\right]\right\} \tag{8}$$

where

$$\mathrm{f}(X_i, P_i) = \arctan(X_i) + i\arctan(P_i) \tag{9}$$

$$\sigma(T_i) = \frac{\Delta\tau}{1 + \exp(-T_i)} \tag{10}$$

and $\mathbf{v} = \{X_i, P_i, T_i : i = 1, \ldots, N\}$ is the set of parameters that defines the pulse. The parameters $\{X_i : X_i \in \mathbb{R}\}$ and $\{P_i : P_i \in \mathbb{R}\}$ are related to the quadrature of the pulse, which is constrained to the interval $[-1, 1]$ by arctan, while the $\{T_i : T_i \in \mathbb{R}\}$ are related to the duration of each segment, which is constrained to the interval $[0, \Delta\tau]$. We fix the number of piecewise-constant intervals, s, as well as the relative unitless time scale $\Delta\tau$.

As elaborated in the main text, this produces a well constrained control pulse with a small number of piecewise-constant intervals (for instance, we used 60 steps). This leads to faster convergence and it is numerically much less taxing than other approaches. However, as a second step we use typical optimal control techniques (like GRAPE) with much higher time-resolution in order to smooth the control pulses. The figures in the main text show this smoothed control pulse, but here in Supplementary Figure 4 and Supplementary Figure 5 we show what the initial piecewise-constant pulses look like. In particular, Supplementary Figure 5 also shows the trajectories for the Toffoli and code-encoding gates, which were described only in prose in the main text.

Lastly, Supplementary Figure 6 and Supplementary Figure 7 showcase the good performance of our parameterization under gradient descent. In particular, we see that as we relax the various regularization constraints (like permitting higher amplitudes or longer durations) we obtain better performing control pulses.

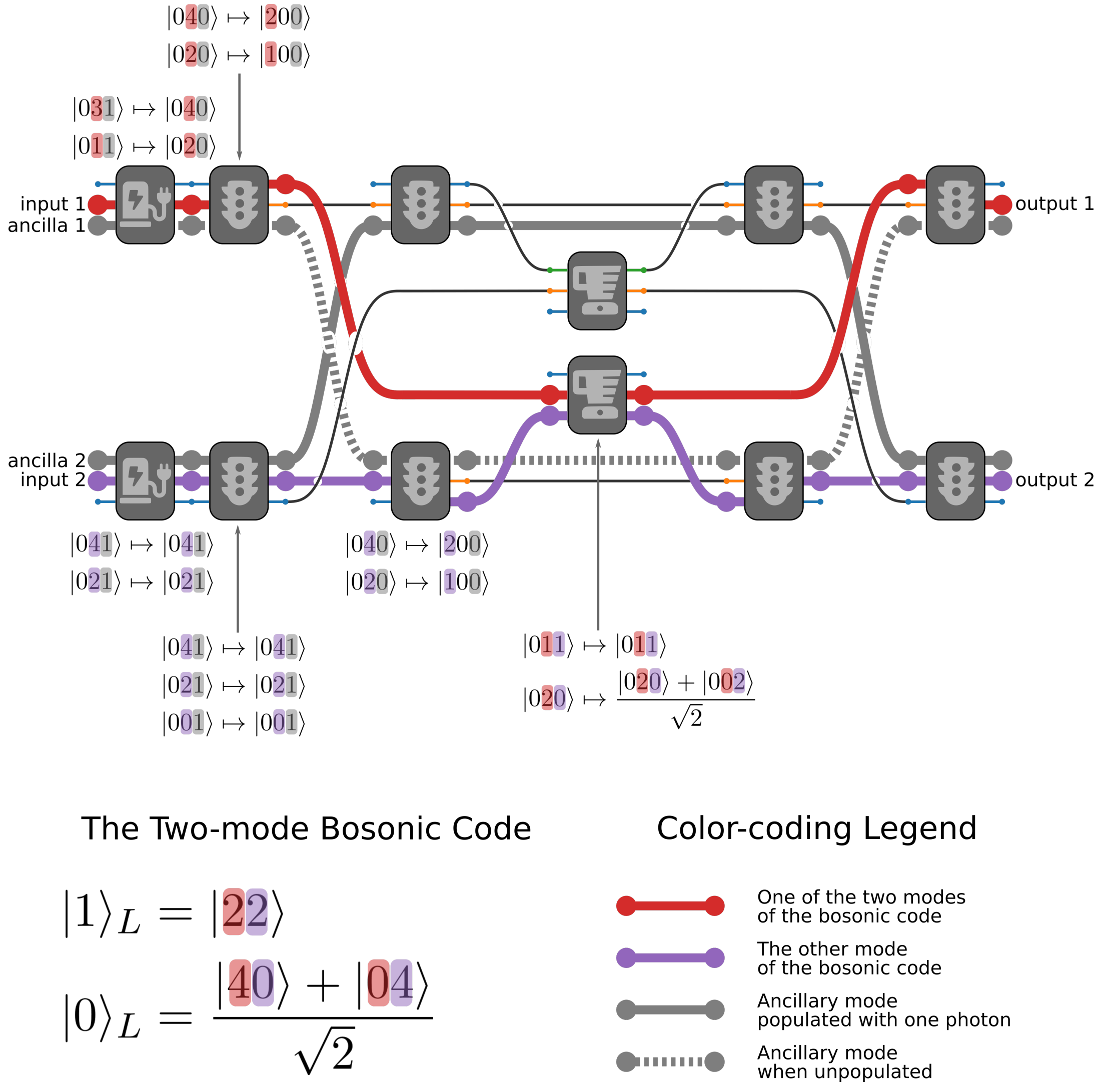

Supplementary Figure 1. A more detailed rendition of Fig. 4, specifically following the trajectory taken by the circuit in the case of a photon loss error on the first of the two modes of the error correcting code (shaded in red). In other words, the input to the circuit is the error state $\alpha|12\rangle + \beta|30\rangle$ and the output is the corrected state $\alpha|22\rangle + \beta\frac{|40\rangle+|04\rangle}{\sqrt{2}}$. Gates are annotated with the unitary operations that they perform. The equation insets showing these operations are color coded to make clear the mapping between available hardware modes and code modes. The gate pictographs are taken from the Font Awesome icon set.

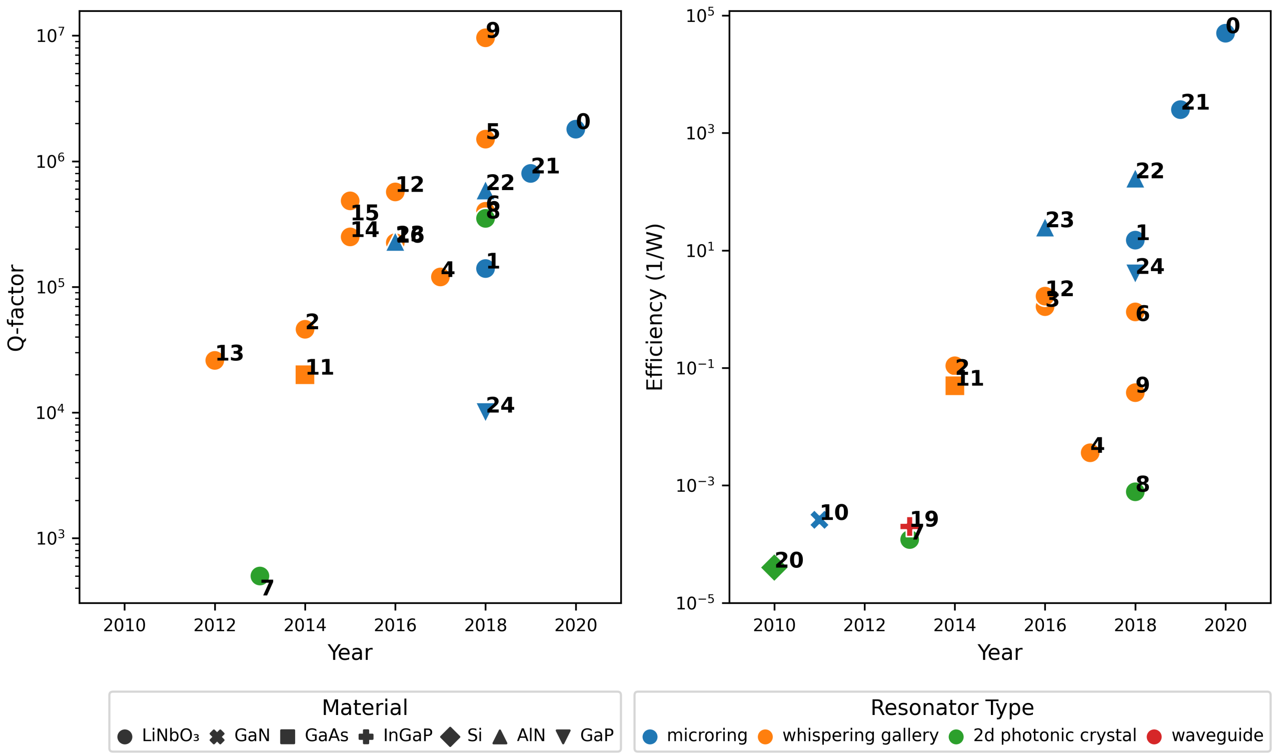

Supplementary Figure 2. Recent progress in SHG experiments. Numerical labels are linked to the corresponding publication in Table I.

	Year	Publication
0	2020	Towards 1% single photon nonlinearity with periodically-poled lithium niobate microring resonators
1	2018	Optical Parametric Generation in a Lithium Niobate Microring with Modal Phase Matching
2	2014	Integrated high quality factor lithium niobate microdisk resonators
3	2016	Phase-Matched Second-Harmonic Generation in an On-Chip LiNbO3 Microresonator
4	2017	On-chip second-harmonic generation and broadband parametric down-conversion in a lithium niobate microresonator
5	2018	High-Q chaotic lithium niobate microdisk cavity
6	2018	Quasi-phase-matched nonlinear optical frequency conversion in on-chip whispering galleries
7	2013	Second harmonic generation in free-standing lithium niobate photonic crystal L3 cavity
8	2018	High-Q two-dimensional lithium niobate photonic crystal slab nanoresonators
9	2018	Highly-efficient second and third harmonic generation in a monocrystalline lithium niobate microresonator
10	2011	Integrated GaN photonic circuits on silicon (100) for second harmonic generation
11	2014	Second-harmonic generation using -quasi-phasematching in a GaAs whispering-gallery-mode microcavity
12	2016	Efficient second harmonic generation in lithium niobate on insulator
13	2012	High-quality LiNbO3 microdisk resonators by undercut etching and surface tension reshaping
14	2015	Fabrication of high-Q lithium niobate microresonators using femtosecond laser micromachining
15	2015	High-Q lithium niobate microdisk resonators on a chip for efficient electro-optic modulation
16	2016	Chip-scale cavity optomechanics in lithium niobate
17	2004	Continuous-wave second-harmonic generation in modal phase matched semiconductor waveguides
18	2005	Efficient continuous wave second harmonic generation pumped at 1.55 μm in quasi-phase-matched AlGaAs waveguides
19	2013	Efficient second harmonic generation in nanophotonic waveguides for optical signal processing
20	2010	Low-power continuous-wave generation of visible harmonics in silicon photonic crystal nanocavities
21	2019	Periodically poled thin-film lithium niobate microring resonators with a second-harmonic generation efficiency of 250,000%/W
22	2018	17000%/W second-harmonic conversion efficiency in single-crystalline aluminum nitride microresonators
23	2016	Second-harmonic generation in aluminum nitride microrings with 2500%/W conversion efficiency
24	2018	400%/W second harmonic conversion efficiency in 14 μm-diameter gallium phosphide-on-oxide resonators

Supplementary Table I. Table of recent SHG experiments featured in Supplementary Figure 2. Each row is hyperlinked to the corresponding publication.

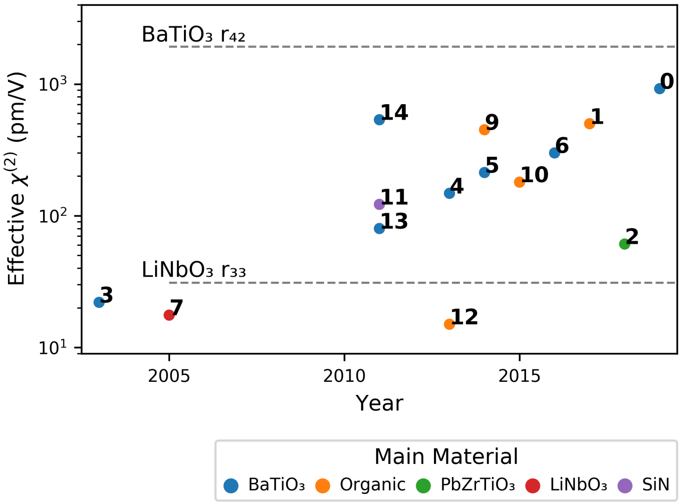

Supplementary Figure 3. Recent experimental progress in Pockels effect electro-optical modulators. This plot showcases significant process in material science and thin-slab fabrication techniques. Numerical labels are linked to the corresponding publication in Table II.

	Year	Publication
0	2019	Large Pockels effect in micro- and nanostructured barium titanate integrated on silicon
1	2017	Silicon–Organic and Plasmonic–Organic Hybrid Photonics
2	2018	Nanophotonic Pockels modulators on a silicon nitride platform
3	2003	Integrated optical Mach–Zehnder modulator based on polycrystalline BaTiO3
4	2013	A strong electro-optically active lead-free ferroelectric integrated on silicon
5	2014	Active Silicon Integrated Nanophotonics: Ferroelectric BaTiO3 Devices
6	2016	A Hybrid Barium Titanate–Silicon Photonics Platform for Ultraefficient Electro-Optic Tuning
7	2005	Electro-optic properties of c-axis oriented LiNbO3 films grown on Si(1 0 0) substrate
8	2011	Hybrid Si-$LiNbO_3$ microring electro-optically tunable resonators for active photonic devices
9	2014	Benzocyclobutene barrier layer for suppressing conductance in nonlinear optical devices during electric field poling
10	2015	All-plasmonic Mach–Zehnder modulator enabling optical high-speed communication at the microscale
11	2011	Pockels effect based fully integrated, strained silicon electro-optic modulator
12	2013	Low Power Mach–Zehnder Modulator in Silicon-Organic Hybrid Technology
13	2011	Ferroelectic BaTiO3 Thin Film Optical Waveguide Modulators
14	2011	Ferroelectic BaTiO3 Thin Film Optical Waveguide Modulators

Supplementary Table II. Table of recent electro-optical modulator experiments featured in Supplementary Figure 3. Each row is hyperlinked to the corresponding publication.

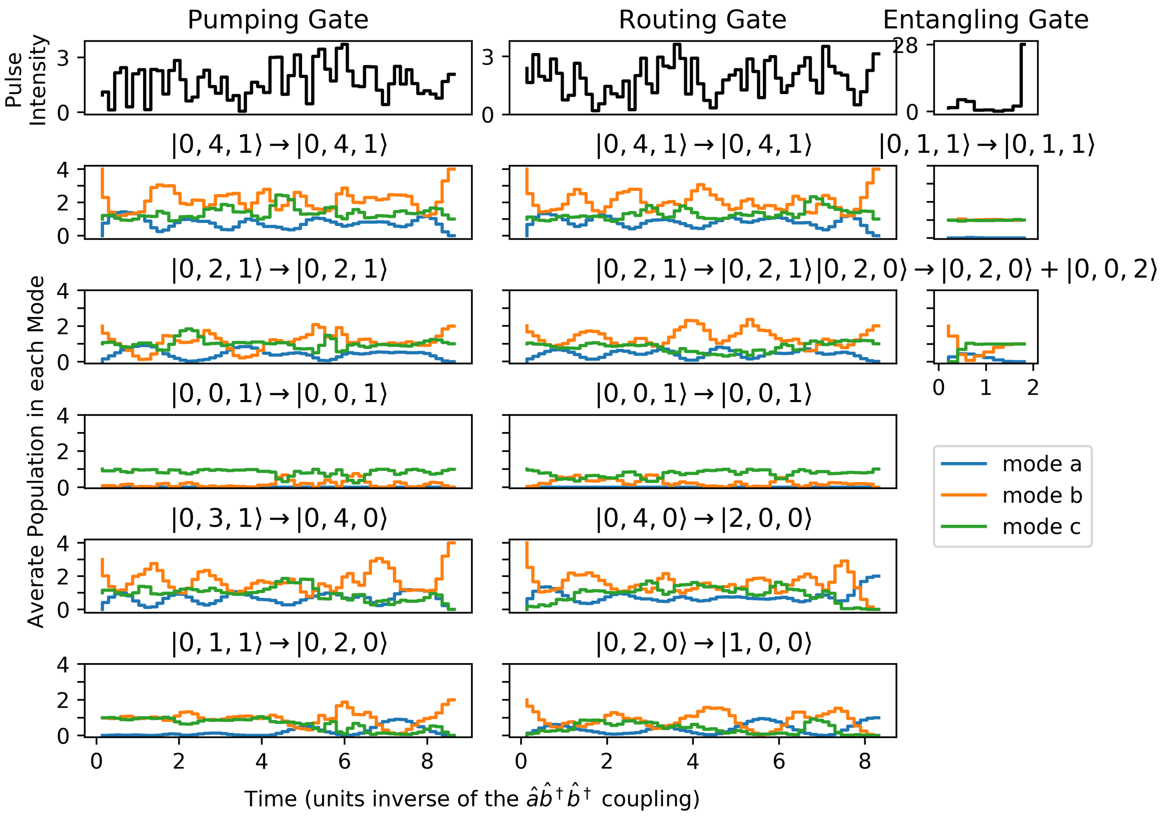

Supplementary Figure 4. The piece-wise constant control pulses before the smoothing stage. This rich piece-wise constant parameterization admits nearly as much freedom as typical high time-resolution characterizations at much lower computational cost. In this particular case we have used 60 time steps of variable width (instead of the hundreds required for a smooth signal).

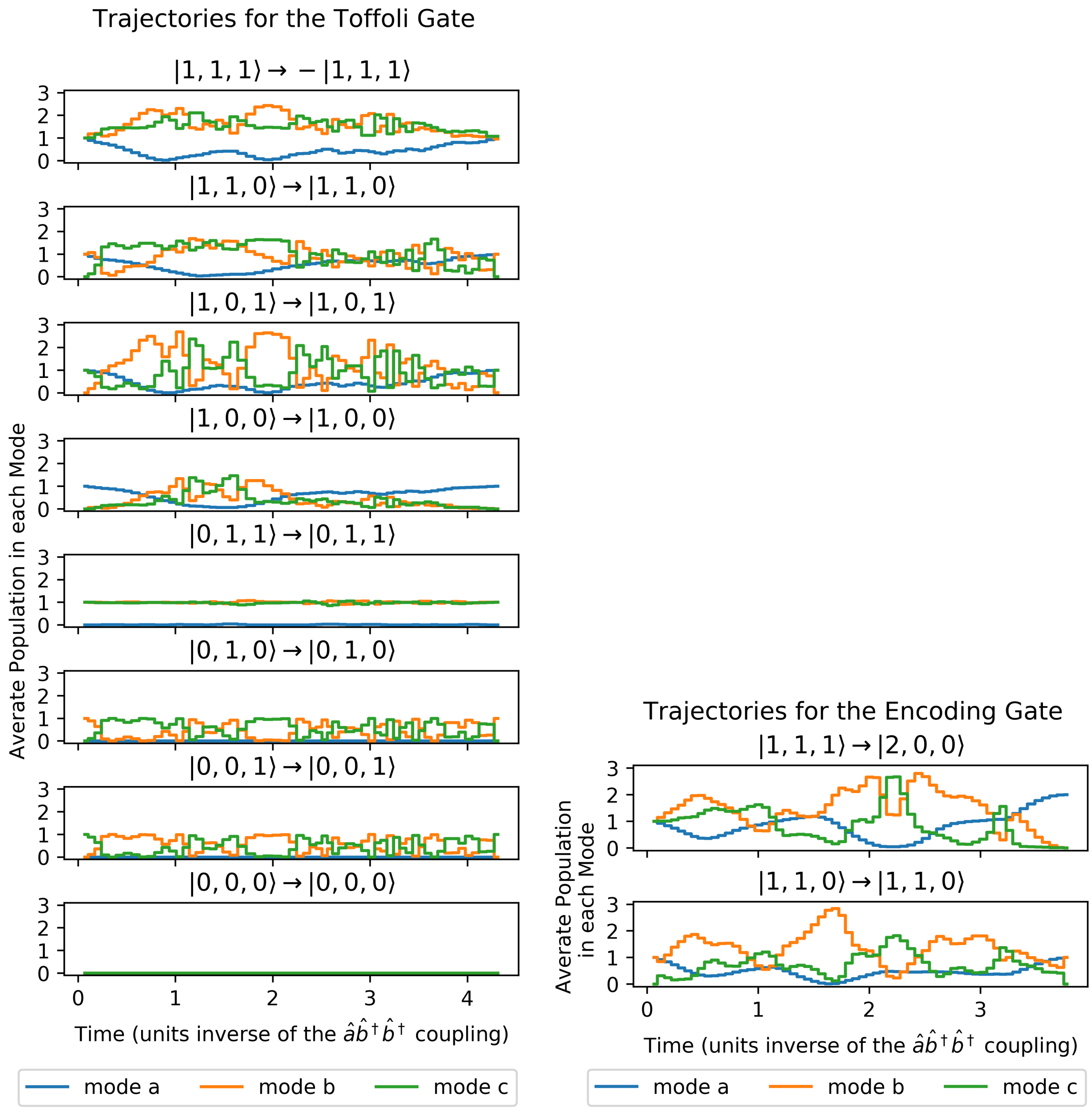

Supplementary Figure 5. The trajectories undergone by various basis states for the Toffoli gate and the code-encoding gate described in the main text.

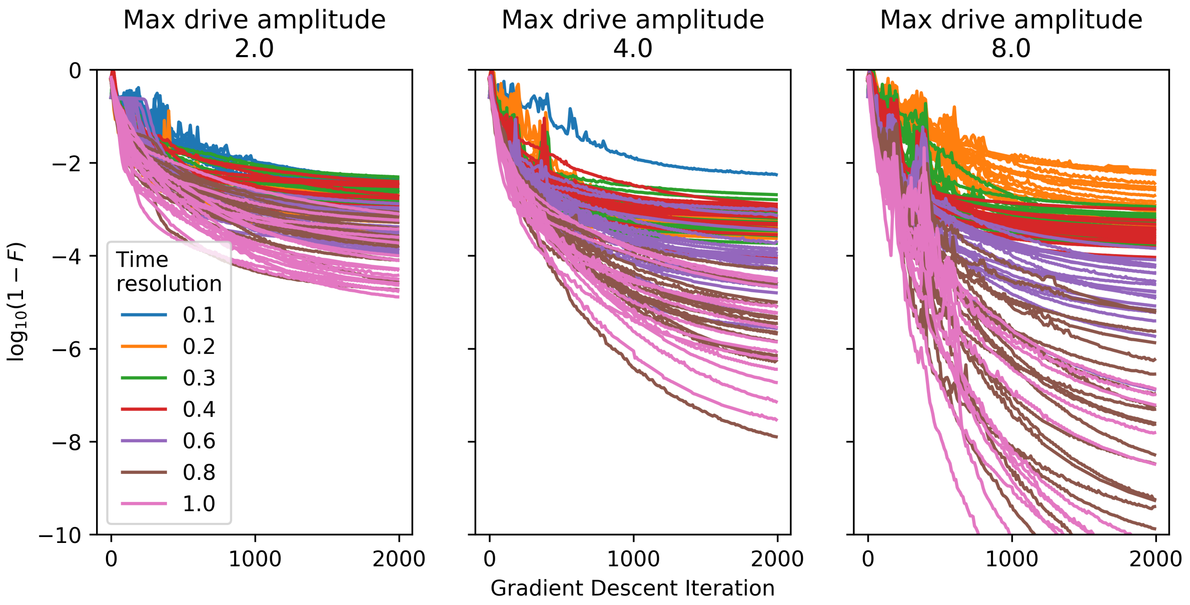

Supplementary Figure 6. The gradient descent optimization procedure performs differently depending on the constraints we have put on our control pulses. The plots above show the infidelity $1 - F$ for a control pulse implementing a particular unitary operation as a function of the iteration steps of the gradient descent optimizer. Each facet depicts different unitless maximum amplitude being permitted for the control drive. Colors represent the unitless characteristic duration $\Delta\tau$ for each piece-wise constant piece of the control drive. We use 60 such pieces in each of the control pulses being considered. There are multiple lines of the same color as we rerun the optimization with varying initial guesses for the control pulse. We see that the gradient descent consistently finds a solution, but plateaus to rather high infidelities of 0.01 if the pulse is overconstrained (e.g. too short or too weak). As we permit higher amplitudes and longer pulses we see that the gradient descent finds much better pulses with infidelities approaching the floating point numerical floor.

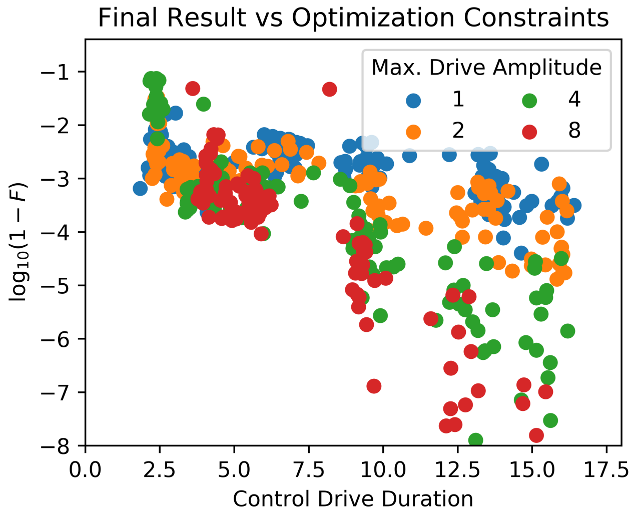

Supplementary Figure 7. This plot shows the same data as Supplementary Figure 6, focusing on the performance of the designed unitary operation after the gradient descent has concluded (i.e. only the points at the 2000th iteration from Supplementary Figure 6). We again see that less constrained pulses (higher permitted amplitude or duration) permit much higher fidelities, approaching the floating point numerical limits. In particular, the infidelity drops exponentially with the length of the pulse. This would be countered with the exponential growth of the chance for photon loss as the pulse duration increases, leading to an optimal pulse duration compromising between the two effects.

SUPPLEMENTARY REFERENCES

[1] Jönsson, M. & Björk, G. Evaluating the performance of photon-number-resolving detectors. *Physical Review A* **99**, 043822 (2019).

[2] Young, S. M., Sarovar, M. & Léonard, F. General modeling framework for quantum photodetectors. *Physical Review A* **98**, 063835 (2018).

[3] Ducci, S. *et al.* Continuous-wave second-harmonic generation in modal phase matched semiconductor waveguides. *Applied physics letters* **84**, 2974–2976 (2004).

[4] Yu, X., Scaccabarozzi, L., Harris, J., Kuo, P. & Fejer, M. Efficient continuous wave second harmonic generation pumped at 1.55 μm in quasi-phase-matched algaas waveguides. *Optics Express* **13**, 10742–10748 (2005).

[5] Galli, M. *et al.* Low-power continuous-wave generation of visible harmonics in silicon photonic crystal nanocavities. *Optics express* **18**, 26613–26624 (2010).

[6] Xiong, C. *et al.* Integrated gan photonic circuits on silicon (100) for second harmonic generation. *Optics express* **19**, 10462–10470 (2011).

[7] Wang, T.-J., He, J.-Y., Lee, C.-A. & Niu, H. High-quality linbo 3 microdisk resonators by undercut etching and surface tension reshaping. *Optics Express* **20**, 28119–28124 (2012).

[8] Diziain, S. *et al.* Second harmonic generation in free-standing lithium niobate photonic crystal l3 cavity. *Applied Physics Letters* **103**, 051117 (2013).

[9] Lenglé, K. *et al.* Efficient second harmonic generation in nanophotonic waveguides for optical signal processing. *Applied Physics Letters* **102**, 151114 (2013).

[10] Kuo, P. S., Bravo-Abad, J. & Solomon, G. S. Second-harmonic generation using-quasi-phasematching in a gaas whispering-gallery-mode microcavity. *Nature communications* **5**, 3109 (2014).

[11] Wang, C. *et al.* Integrated high quality factor lithium niobate microdisk resonators. *Optics express* **22**, 30924–30933 (2014).

[12] Lin, J. *et al.* Fabrication of high-q lithium niobate microresonators using femtosecond laser micromachining. *Scientific reports* **5**, 8072 (2015).

[13] Wang, J. *et al.* High-q lithium niobate microdisk resonators on a chip for efficient electro-optic modulation. *Optics express* **23**, 23072–23078 (2015).

[14] Moore, J. *et al.* Efficient second harmonic generation in lithium niobate on insulator. In *2016 Conference on Lasers and Electro-Optics (CLEO)*, 1–2 (IEEE, 2016).

[15] Lin, J. *et al.* Phase-matched second-harmonic generation in an on-chip l i nbo 3 microresonator. *Physical Review Applied* **6**, 014002 (2016).

[16] Luo, R. *et al.* On-chip second-harmonic generation and broadband parametric down-conversion in a lithium niobate microresonator. *Optics express* **25**, 24531–24539 (2017).

[17] Wang, L. *et al.* High-q chaotic lithium niobate microdisk cavity. *Optics letters* **43**, 2917–2920 (2018).

[18] Wolf, R. *et al.* Quasi-phase-matched nonlinear optical frequency conversion in on-chip whispering galleries. *Optica* **5**, 872–875 (2018).

[19] Li, M., Liang, H., Luo, R., He, Y. & Lin, Q. High-q two-dimensional lithium niobate photonic crystal slab nanoresonators. *arXiv preprint arXiv:1806.04755* (2018).

[20] Lin, J. *et al.* Highly-efficient second and third harmonic generation in a monocrystalline lithium niobate microresonator. *arXiv preprint arXiv:1809.04523* (2018).

[21] Luo, R. *et al.* Optical parametric generation in a lithium niobate microring with modal phase matching. *Physical Review Applied* **11**, 034026 (2019).

[22] Lu, J. *et al.* Periodically poled thin-film lithium niobate microring resonators with a second-harmonic generation efficiency of 250,000%/w. *Optica* **6**, 1455–1460 (2019).

[23] Bruch, A. W. *et al.* 17 000%/w second-harmonic conversion efficiency in single-crystalline aluminum nitride microresonators. *Applied Physics Letters* **113**, 131102 (2018).

[24] Guo, X., Zou, C.-L. & Tang, H. X. Second-harmonic generation in aluminum nitride microrings with 2500%/w conversion efficiency. *Optica* **3**, 1126–1131 (2016).

[25] Logan, A. D. *et al.* 400%/w second harmonic conversion efficiency in 14 μm-diameter gallium phosphide-on-oxide resonators. *Optics express* **26**, 33687–33699 (2018).

[26] Petraru, A., Schubert, J., Schmid, M., Trithaveesak, O. & Buchal, C. Integrated optical mach zehnder modulator based on polycrystalline batio 3. *Optics letters* **28**, 2527–2529 (2003).

[27] Akazawa, H. & Shimada, M. Electro-optic properties of c-axis oriented linbo3 films grown on si (1 0 0) substrate. *Materials Science and Engineering: B* **120**, 50–54 (2005).

[28] Lee, Y. S. *et al.* Hybrid si-linbo 3 microring electro-optically tunable resonators for active photonic devices. *Optics letters* **36**, 1119–1121 (2011).

[29] Chmielak, B. *et al.* Pockels effect based fully integrated, strained silicon electro-optic modulator. *Optics express* **19**, 17212–17219 (2011).

[30] Petraru, A., Siegert, M., Schmid, M., Schubert, J. & Buchal, C. Ferroelectic batio 3 thin film optical waveguide modulators. *MRS Online Proceedings Library Archive* **688** (2001).

[31] Abel, S. *et al.* A strong electro-optically active lead-free ferroelectric integrated on silicon. *Nature communications* **4**, 1671 (2013).

[32] Palmer, R. *et al.* Low power mach–zehnder modulator in silicon-organic hybrid technology. *IEEE Photonics Technology Letters* **25**, 1226–1229 (2013).

[33] Xiong, C. *et al.* Active silicon integrated nanophotonics: ferroelectric batio3 devices. *Nano letters* **14**, 1419–1425 (2014).

[34] Jin, W. *et al.* Benzocyclobutene barrier layer for suppressing conductance in nonlinear optical devices during electric field poling. *Applied Physics Letters* **104**, 94_1 (2014).

[35] Haffner, C. *et al.* All-plasmonic mach–zehnder modulator enabling optical high-speed communication at the microscale. *Nature Photonics* **9**, 525 (2015).

[36] Abel, S. *et al.* A hybrid barium titanate–silicon photonics platform for ultraefficient electro-optic tuning. *Journal of Lightwave Technology* **34**, 1688–1693 (2016).

[37] Heni, W. *et al.* Silicon–organic and plasmonic–organic hybrid photonics. *ACS Photonics* **4**, 1576–1590 (2017).

[38] Alexander, K. *et al.* Nanophotonic pockels modulators on a silicon nitride platform. *Nature communications* **9**, 3444 (2018).

[39] Abel, S. *et al.* Large pockels effect in micro-and nanostructured barium titanate integrated on silicon. *Nature materials* **18**, 42 (2019).
